# Cracking Characteristics of Asphalt Pavement Under Thermal Stresses

**DOI:** 10.3390/ma19040771

**Published:** 2026-02-16

**Authors:** Jingwei Jia, Mengfan Zhang, Jinxi Zhang, Chao Jing

**Affiliations:** 1 Qinhuangdao Institute of Quality and Technical Supervision and Inspection, Qinhuangdao 066000, China; 18503317335@163.com; 2 Hebei Key Laboratory of Green Construction and Intelligent Maintenance for Civil Engineering, Yanshan University, Qinhuangdao 066000, China; zwnaqjdc@163.com; 3 Zhangweinan Canal Administration Bureau, Dezhou 253000, China; 4 Beijing Key Laboratory of Traffic Engineering, Beijing University of Technology, Beijing 100011, China; zhangjinxi@bjut.edu.cn; 5 Beijing Engineering Research Center of Urban Transport Operation Guarantee, Beijing University of Technology, Beijing 100011, China; 6 Hebei Province Engineering Research Center for Harmless Synergistic Treatment and Recycling of Municipal Solid Waste, Yanshan University, Qinhuangdao 066000, China

**Keywords:** asphalt pavement, thermal stresses, cracking characteristics, pavement life

## Abstract

To evaluate the cracking characteristics of asphalt pavements under thermal stresses, the finite element (FE) software ABAQUS 2021 was used in this paper to establish thermal and mechanical parameter models, respectively. The temperature field distributions in winter and summer were analyzed according to the actual situation based on fracture mechanics theory and the extended FE method, as well as the most unfavorable crack type for crack propagation was also studied. Further, the impact of the propagation of transverse cracks on the road surface was investigated by changing the solar radiation, sunshine duration, and wind speed. Finally, the propagation pattern of reflective cracks was observed under the cyclic temperature field. The results show that under the action of the temperature field alone, type I cracks, which are cracks that undergo opening displacement due to the vertical tensile stress acting on the crack surface, are the main type of cracks, while the trend of crack propagation was much higher in winter than in summer. It was also found that changing the parameters of solar radiation, sunshine duration, and wind speed could significantly impact cracking. Under the cyclic temperature field, the length of reflective cracks was proportional to time, and the initial crack length significantly affected the pavement life. Therefore, pavement inspection should be more stringent in winter, and initial cracks should be avoided as much as possible during paving.

## 1. Introduction

With rapid advancement and economic development, China has made significant achievements in the transportation industry, attracting global attention. Today, China’s transportation infrastructure has been completed, spreading the transportation network throughout the country. However, pavements experience many environmental issues during service life due to significant seasonal temperature variations and great climate disparities among regions. In particular, the significant temperature differences between day and night, especially in northern China, are more prone to cause pavement shrinkage. When shrinkage stresses exceed the strength of asphalt pavements, cracking may occur. Furthermore, a temperature gradient is formed among different structural layers of the pavement due to the disparity in their thermal expansion coefficients [1], resulting in different levels of stress and deformation between the structural layers, which eventually cause pavement cracking.

**Figure 1:**
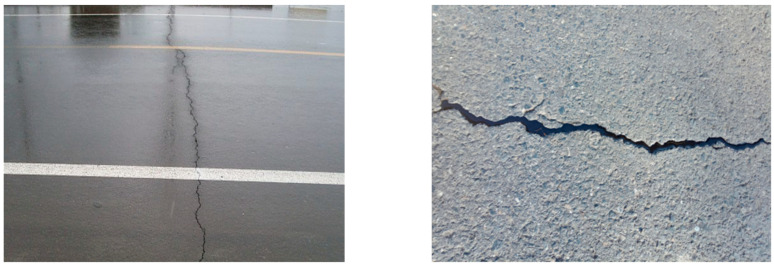
Top-down transverse cracks on the road surface and bottom-up reflective cracks on the base layer.

There are many forms of cracks occurring in flexible pavements. The most common ones are top-down transverse cracks and bottom-up reflective cracks, as shown in Figure 1. In the surface layer, temperature-induced cracks mainly appear as transverse cracks. Once the transverse cracks are formed, they continue to expand downward under the action of temperature, forming top-down cracks and eventually leading to the complete failure of the asphalt pavement [2]. The crack tip is susceptible to local stress concentration of the base layer under cyclic temperature loads. When the stress exceeds a certain level, the crack passes through the base layer and expands upward, eventually reaching the entire surface layer.

Thermal stress-induced cracking in asphalt pavements has drawn extensive research attention, as it significantly affects pavement durability and structural performance. Lian et al. [3] highlighted that the stress and strain states of semi-rigid base layers are highly sensitive to ambient temperature and humidity, and prolonged exposure to such variations can lead to cumulative structural damage. Building upon that, Wu et al. [4] investigated thermal-cracking behavior using semi-circular bending tests and derived key parameters, further examining the combined effects of temperature and other factors on crack growth. Beyond thermal loading, Zha et al. [5] employed ABAQUS to assess damage in different pavement structures under blast loads, while Chen et al. [6] developed a thermo-mechanical coupling model validated with experimental data to analyze crack-related distress in bridge deck pavements. At the microscale, Xia et al. [7] established a three-phase mesoscopic model to explore crack initiation and propagation mechanisms in asphalt concrete.

In the context of reflective cracking, Dave et al. [8] adopted a viscoelastic modeling approach and demonstrated that base cracks remain stable during temperature rise but propagate during cooling cycles. Similarly, Hossain et al. [9] utilized the extended finite element method (XFEM) to simulate crack growth under thermal fatigue, and Gallego et al. [10] designed a laboratory device to evaluate wheel- and temperature-induced reflective cracking, enabling direct measurement of thermal-contraction displacements. Han et al. [11] performed a two-dimensional temperature-field analysis of pavements with three subgrade types using ABAQUS, offering improved accuracy for pavement damage assessment. Through field experiments at airports, Ji et al. [12] reported that unloaded cracks predominantly result from moisture loss or temperature fluctuations.

Regarding low-temperature cracking, Cannone et al. [13] and Braham et al. [14] conducted comparative evaluations between experimental measurements and numerical simulations under indirect tensile, flexural, and single-edge notched beam tests. Timm et al. [15] identified through field measurements that crack spacing follows predictable patterns. Xue et al. [16] evaluated the stress-field distribution in asphalt pavements under varying temperatures by incorporating convective heat transfer, thermal radiation, and thermoviscoelastic deformation theories. The studies by Zheng et al. [17] and Krishnan et al. [18] further corroborated that pavement temperature fluctuations significantly alter the physical parameters of asphalt layers. Baek, Kim, Ban et al. [19] quantitatively assessed cracking at low temperatures by varying pavement geometry and material properties.

Concerning crack geometry and numerical modeling, Huang et al. [20] developed finite-element models with multiple crack inclinations to analyze orientation-dependent cracking behavior. Wang et al. [21] combined temperature-field simulation with XFEM to study crack mechanisms and propagation patterns under cyclic thermal loading. Kim [22] constructed both 2D and 3D pavement finite-element models incorporating a nonlinear constitutive description of asphalt mixtures, revealing that reducing the temperature gradient can effectively retard reflective-crack propagation. Moreover, a series of numerical studies have contributed to the understanding of crack-propagation mechanisms under thermal stresses [23,24,25,26].

Despite these advances, several research gaps remain:

(1) Most existing models focus either on a single crack type (e.g., reflective cracks) or assume idealized temperature conditions, leaving a comparative analysis of different crack-initiation modes—particularly top-down versus bottom-up cracking—under realistic spatiotemporally varying temperature fields largely unexplored.
(2) The majority of thermal-crack coupling studies rely on simplified two-dimensional frameworks, which cannot fully capture the interaction between three-dimensional transient temperature fields and existing cracks.
(3) Although methods such as XFEM and thermo-mechanical coupling have been introduced, an integrated approach that combines transient heat-transfer theory with fracture mechanics to systematically evaluate the effects of multiple environmental parameters—solar radiation, wind speed, and exposure duration—on crack-propagation rates and modes is still lacking.

To address these limitations, this study investigates the influence of temperature on pavement cracking using two distinct three-dimensional finite-element models developed in ABAQUS 2021, representing top-down and bottom-up crack scenarios. The models are founded on coupled heat-transfer and linear-elastic fracture-mechanics theories, enabling the simulation of transient temperature fields and the subsequent computation of thermal stress intensity factors. By parameterizing key environmental variables--including solar radiation, wind speed, and exposure duration--this work aims to systematically quantify their impacts on crack propagation, thereby providing a more comprehensive framework for assessing thermal-cracking susceptibility in asphalt pavements.

## 2. Temperature Field Modeling

### 2.1. Temperature Field Theory

Due to solar radiation, the atmospheric temperature has noticeable differences during day and night and shows diurnal periodic variation characteristics. The influence of the periodic variation in solar radiation on the temperature field of the pavement structure can be approximated by periodically changing boundary conditions [27]. For semi-rigid base asphalt pavements, many factors affect the temperature field, such as total solar radiation, wind speed, effective sunshine, and exposure duration. In this paper, the boundary conditions of pavements under periodic temperature fields are presented in the following three aspects.

#### 2.1.1. Solar Radiation

The diurnal variation process of solar radiation can be approximately described by Equation (1) [28].

(1) $$q(t)=\left\{\begin{array}{ll} 0 & 0\leq t\leq 12-\frac{c}{2}\\ q_{0}cosm\omega (t-12) & 12-\frac{c}{2}\leq t\leq 12+\frac{c}{2}\\ 0 & 12+\frac{c}{2}\leq t\leq 24 \end{array}\right.$$

where $q_{0}$ (J/m^2^) is the maximum solar radiation at noon, $q_{0}=0.131mQ$, m=12/c; Q (J/m^2^) is the daily total solar radiation; c (h) is the number of actual effective sunshine hours; ω (rad) is the angular frequency, ω=2π/24.

Since the aforementioned piecewise function (Equation (1)) has discontinuities, it needs to be expanded as a series and converted into a smooth continuous form of the cosine function through the Fourier transform, as given in Equation (2).

(2) $$q(t)=\frac{a_{0}}{2}+\sum_{k=1}^{\infty }\;a_{k}cos\frac{k\pi (t-12)}{12}$$

where

(3) $$\begin{array}{c} a_{0}=\frac{2q_{0}}{m\pi }\;\;\;\;\;\;\;\;\;\;\;\;\;\;\;\;\;\;\;\;\;\;\;\;\;\;\;\;\;\;\;\;\;\;\;\;\;\;\;\;\;\;\;\;\;\;\;\;\;\;\;\;\;\;\;\;\;\;\;\;\;\;\;\;\;\;\;\;\;\;\;\\ a_{k}=\left\{\begin{array}{l} \frac{q_{0}}{\pi }\left[\frac{1}{m+k}sin(m+k)\frac{\pi }{2m}+\frac{\pi }{2m}\right]\\ \frac{q_{0}}{\pi }\left[\frac{1}{m+k}sin(m+k)\frac{\pi }{2m}+\right.\\ \left.\frac{1}{m-k}sin(m-k)\frac{\pi }{2m}\right] \end{array}\;k\neq m\right. \end{array}$$

#### 2.1.2. Air Temperature and Convective Heat Exchange

Like solar radiations, atmospheric temperature also follows a periodic pattern. The lowest temperature of the day occurs at around 5 a.m. and the highest around 2 p.m., indicating that the warming duration (i.e., 9 h) is much shorter than the cooling duration (i.e., 15 h). Therefore, only a single sine function cannot accurately describe the convective exchange of temperature. The condition is more realistic when the double sine function is combined into a new linear expression, as given in Equation (4).

(4) $$T_{a}={\bar{T}}_{a}+T_{m}\left[0.96sin\omega \left(t-t_{0}\right)+0.14sin2\omega \left(t-t_{0}\right)\right]$$

where ${\bar{T}}_{a}$ is the daily average temperature, ${\bar{T}}_{a}=\frac{1}{2}\left(T_{a}^{max}+T_{a}^{min}\right)$; $T_{m}$ is the daily temperature variation, $T_{m}=\frac{1}{2}\left(T_{a}^{max}-T_{a}^{min}\right)$; $T_{a}^{max}$ and $T_{a}^{min}$ are the daily maximum temperature and the daily minimum temperature, respectively; ω (rad) is the angular frequency, ω=2π/24; $t_{0}$ is the initial phase, generally $t_{0}=9$.

The heat exchange coefficient $h_{c}$ of the heat exchange between the road surface and the atmosphere is mainly related to the wind speed $v_{\omega }$. The linear correlation of $h_{c}$ and $v_{\omega }$ is expressed in Equation (5).

(5) $$h_{c}=3.7v_{\omega }+9.4$$

where $h_{c}$ is the heat exchange coefficient $W/\left(m^{2}\;{}^{\circ}C\right)$; $v_{\omega }$ is the average daily wind speed (m/s).

#### 2.1.3. Effective Radiation of the Pavement

The effective radiation of the pavement is related to air temperature, cloud cover, air humidity, and transparency. Many studies have suggested using the method of changing the heat transfer coefficient of the road surface to correct the air temperature. However, this method has significant limitations. Thus, Equation (6) is used to realize the boundary conditions of the effective ground surface radiation.

(6) $$q=\varepsilon \sigma \left[{\left({\left.T_{l}\right|}_{z=0}-T_{z}\right)}^{4}-{\left(T_{a}-T_{z}\right)}^{4}\right]$$

where $q_{F}$ is the effective ground surface radiation; γ is the emissivity of the pavement, 0.81 for asphalt pavement; σ is the Stefan-Boltzmann constant, $5.6697\times 10^{-8}\;W\;\left(m^{2}{\;K}^{4}\right)$; ${\left.T_{l}\right|}_{z=0}$ is the road surface temperature; $T_{a}$ is the atmospheric temperature; $T_{z}$ is the absolute zero, $T_{z}=-273\;{}^{\circ}C$.

### 2.2. Thermodynamic Modeling of the Asphalt Pavement

Considering the boundary effect of the model and the actual analysis speed of the software (ABAQUS 2021), a three-dimensional FE model of 6 m × 6 m × 3 m was created in this study. The main structure was divided into three layers, i.e., surface layer, base layer, and soil base. The surface layer was divided into upper, middle, and lower layers, with 4 cm, 6 cm, and 10 cm thicknesses, respectively. The thickness of the base layer is set to 54 cm. A transverse crack, with a length of 4 cm, running through the entire road surface in the middle of the upper layer was placed. Due to the complexity of the actual road environment, the following assumptions were made for developing the FE model.

(1) A viscoelastic constitutive model is adopted for the surface layer material, and the linear elastic constitutive model is used for the base layer and soil base.
(2) The model is entirely continuous between layers. The temperature and heat flow are continuous, and the model is fully bonded between layers.
(3) The heat conduction mode of the model is one-dimensional vertical heat transfer. The solar radiation surface is the upper surface of the asphalt surface layer, and the other surfaces of the model will not be affected by solar radiation.
(4) The influence of road self-weight is ignored in the analysis process.

Among them, assumption (1) assumes that the surface layer adopts a viscoelastic constitutive model to better reflect the actual situation, while the base layer adopts a linear elastic constitutive model because its mechanical properties do not change significantly within the temperature variation range, and using linear elasticity can greatly simplify the calculation; assumption (2) is to ensure that the temperature field can seamlessly conduct from the road surface to the base layer; assumption (3) is to reduce the complexity of the model; assumption (4) is to ignore additional factors and focus the analysis on the temperature changes.

After meshing, the FE model has 201,960 elements, and the element type is an eight-node linear heat transfer hexahedral element (D3D8R). The eight-node linear hexahedral element with reduced integration (C3D8R) was used in the subsequent stress analysis. The .odb file obtained from the thermal model analysis was imported into the mechanical model to continue the stress analysis. In the stress analysis, boundary conditions were applied to constrain the model; the bottom surface of the model was completely fixed, and the horizontal displacements of the remaining four sides were also fixed.

### 2.3. Selecting Thermodynamic Parameters of Asphalt Pavement

The specific thermodynamic parameters between the layers were selected from the results of Gao et al. [29] and are presented in Table 1. The temperatures on a particular day in January and July in an eastern city were selected for analysis. It was assumed that the temperature changes within 30 days of the month follow the same pattern as the selected representative temperature. The impact on the asphalt pavement under typical representative temperatures in winter and summer was then observed. The stress variation in the asphalt pavement was analyzed based on the daily total solar radiation Q, sunshine duration c, and average wind speed v change. The temperature data for summer and winter were obtained from the actual temperature reported by the meteorological bureau of a certain city in the eastern part of our country, as shown in Table 2 and Table 3.

## 3. Influence of Temperature Load on Lateral Crack Propagation on Pavement Surface

### 3.1. Analysis of the Temperature Field Model of Asphalt Pavement

**Figure 2:**
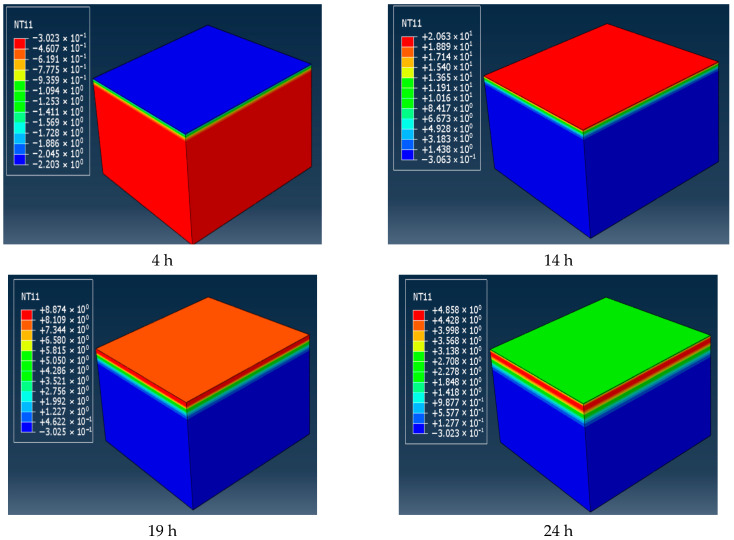
Temperature field distribution at four different moments in 24 h in winter (°C).

The parameter values listed in Table 1, Table 2 and Table 3 were imported into the model through DFLUX and FILM subroutines, and the temperature changes over 24 h in winter and summer could be obtained. The temperature changes in four moments in winter were selected, as shown in Figure 2. As can be seen from the graph, the real-time temperature of the road surface is not consistent with the atmospheric temperature at that moment. This is because the heat capacity and heat conduction efficiency of the atmosphere are different from those of the road surface.

**Figure 3:**
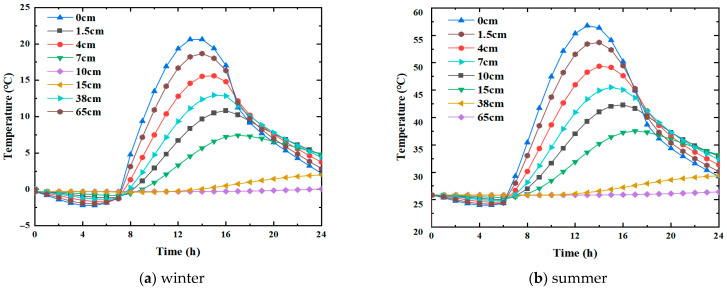
Temperature field variation at different depths.

It is seen that the trends of temperature change in winter and summer are the same, and they all show an overall trend of first rising and then falling. Nevertheless, the corresponding values at each moment are different. Since the modeling assumption was one-dimensional vertical heat transfer, the temperature within the same horizontal pavement layer was the same. In order to facilitate temperature extraction, the middle position of the crack was taken as the research object in this paper, and the corresponding temperature curves at different depths were plotted, as shown in Figure 3.

The analysis of the temperature field variation at different depths in winter and summer indicates the following observations.

(1) Vertically, the temperature change on the road surface is the most significant, whether in winter or summer. In winter, the maximum temperature difference occurred at the road surface, $T_{max}=20.63\;{}^{\circ}C$, $T_{min}=-2.21\;{}^{\circ}C$, i.e., a difference of 22.84 °C. At 38 cm depth, $T_{max}=1.99\;{}^{\circ}C$, $T_{min}=-0.36\;{}^{\circ}C$—the difference being merely 2.35 °C. In summer, the maximum temperature difference also appeared at the road surface, $T_{max}=56.36\;{}^{\circ}C$, $T_{min}=23.99\;{}^{\circ}C$, i.e., the difference is 31.37 °C. At 38 cm depth, $T_{max}=29.36\;{}^{\circ}C$, $T_{min}=25.79\;{}^{\circ}C$, i.e., there was merely a difference of 3.57 °C. It is, thus, inferred that external temperature conditions significantly impact the asphalt pavement, especially the upper and middle layers; however, the impact on the lower layer is smaller, and there is almost no impact on larger depths.
(2) Horizontally, the moments of peak temperature indicate temperature propagation lag, and the moments of peak temperature are not the same at different depths. In winter, the maximum temperature appears at 14 h when the depth is 0 cm. As the depth increases, the moment of maximum temperature moves backward. For example, the maximum temperature appeared at 15 h when the depth was 7 cm, whereas at a depth of 15 cm, it appeared at 17 h. In summer, the maximum temperature also occurred at 14 h when the depth was 0 cm, while the pattern was about the same as in winter.

In summary, the magnitude of temperature change on the surface layer is quite significant, both horizontally and vertically. The transverse crack was located right in the surface layer, so a large temperature stress was generated there, significantly influencing the crack propagation. Most of the analyses were performed on the crack located in the surface layer, as detailed in the following sections.

### 3.2. Effect of Temperature Stresses on Crack Propagation

After obtaining the temperature field model, it was deemed necessary to introduce the thermal expansion coefficient (Williams–Landel–Ferry (WLF) equation parameters) to analyze the effect of temperature on cracks further. The relevant parameters in this study are adopted from the work of Xu et al. [30]. Since the thermal expansion coefficients of materials are closely related to temperature in practice, to reflect the actual situation, the thermal expansion coefficients(×10^−5^/°C) of the surface layer in this paper were set as temperature-dependent parameters and fixed for the base layer and soil base. The specific parameters are listed in Table 4.

The surface layer of the pavement was modeled with a viscoelastic material. Since the mechanical properties of viscoelastic material exhibit remarkable differences at different temperatures, the generalized Maxwell model was introduced in this paper to characterize the viscoelasticity of the surface layer. In the viscoelasticity setup of ABAQUS, the domain was set as time, and the time was changed to Prony. Asphalt mixtures are generally characterized by the time–temperature equivalence principle and fitted by the WLF equation (lgα_T_ = −[C_1_ (T − T_0_)]/[C_2_ + (T − T_0_)]). In the formula, C_1_ and C_2_ are material constants. The corresponding parameters related to the Prony series, asphalt pavement, and WLF equation are given in Table 5, Table 6, and Table 7, respectively.

**Figure 4:**
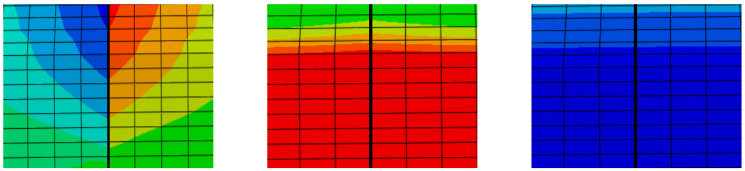
Three-dimensional displacement distribution of the transverse crack in the surface layer of the pavement at a certain moment.

The fracture mechanics theory classifies cracks into three main types: open type (Type I), sliding type (Type II), and tearing type (Type III). The definition of Type I cracks has been stated previously and will not be repeated here. Type II cracks refer to those subjected to shear stress, where the crack edges undergo relative sliding, and the sliding direction is consistent with the shear stress direction. Type III cracks refer to those subjected to shear stress, where the outer layers on both sides of the crack tear each other, and the expansion direction is perpendicular to the shear stress direction. Under the influence of the temperature field, the pavement undergoes a certain degree of shrinkage or expansion with the change in the temperature field. The displacement distribution in the three directions at a typical moment in winter was extracted and plotted separately, as shown in Figure 4. The left figure shows the displacement distribution in the U1 direction. At this moment, the displacement on the left is negative, while that on the right is positive, and the symmetry of the displacement distribution indicates that the inside of the crack is subjected to considerable longitudinal tensile stress. The middle and right figures show that the displacement field at this moment has not changed significantly, indicating that when the cracks are under a varying temperature field, the transverse cracks in the surface layer of the pavement are mainly subjected to longitudinal tensile stress, whereas the shear stress is almost negligible. Therefore, when studying the stress state of cracks in the following sections, the mode I crack, i.e., the opening mode, is the dominant one, while the impact of mode II and III cracks is small and can be ignored.

**Figure 5:**
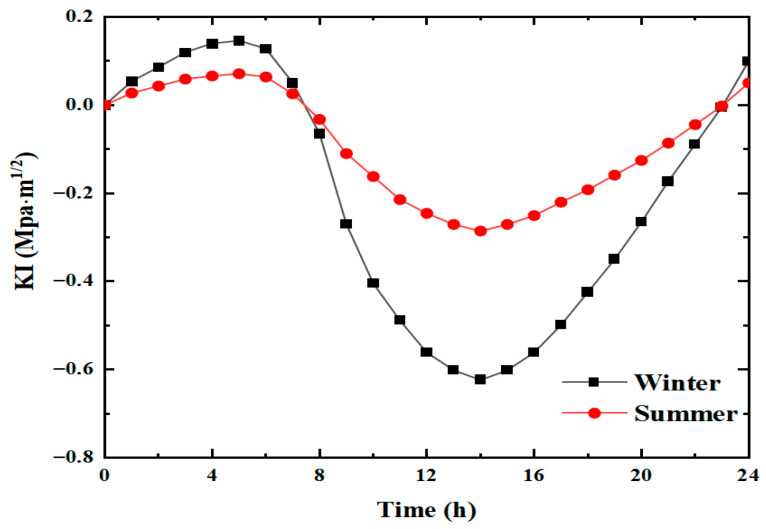
Variation in mode I strength intensity factor in winter and summer.

Since temperature variations in winter and summer are quite significant, exploring their impact on transverse cracks is necessary to identify the most unfavorable season. Figure 5 shows the curves of the mode I stress intensity factor extracted at the crack tip as a function of time. It should be noted that in ABAQUS, the stress values at the nodes near the crack tip and the distance values from the crack tip can be output. When multiple points are output, corresponding distribution curves can be fitted. By using the least squares method for fitting and then calculating the partial derivatives, the corresponding KI values can be obtained.

The variation trend for the mode I strength intensity factor for both winter and summer is similar, i.e., increasing first, then decreasing, and finally increasing again. From 0 h to 8 h in both summer and winter, the mode I stress intensity factor K1 is positive, indicating that the crack tends to open under tension. Starting from 8 h, K1 becomes negative and further decreases until 14 h, manifesting compressive shrinkage. From 14 h to 24 h, although the value of the stress intensity factor rises, it is still negative, so the crack still tends to shrink under compression at this stage. From 23 h to 24 h, K1 becomes positive; at this time, the crack continues to show the trend of tensile opening.

The amplitude of change in K1 is large for both winter and summer. However, the overall magnitude of change in winter is larger than in summer. For winter, the positive peak of K1 of value $0.145\;MPa\cdot m^{1/2}$ appeared at about 5 h, whereas the negative peak was $-0.623\;MPa\cdot m^{1/2}$ occurring at around 14 h—the absolute value of the difference between the two was $0.768\;MPa\cdot m^{1/2}$. For summer, the positive peak of K1 also appeared at about 5 h, which was $0.073\;MPa\cdot m^{1/2}$; however, the corresponding negative peak was $-0.32\;MPa\cdot m^{1/2}$ occurring at around 14 h, and the absolute difference was $0.393\;MPa\cdot m^{1/2}$.

The stress intensity factor variation in the two seasons shows that the magnitude of change in the mode I stress intensity factor in winter is almost twice that in summer. The positive and negative extreme values in winter are much larger than in summer. Therefore, the stress concentration at the crack tip is more significant in winter, and cracks are more likely to develop. Consequently, the winter temperature variation was used to analyze the crack stress state in the subsequent sections.

## 4. Effects of Different Parameters on Crack Propagation Under Winter Temperature Load

### 4.1. Effect of Daily Solar Radiation on Transverse Crack Propagation in the Pavement

**Figure 6:**
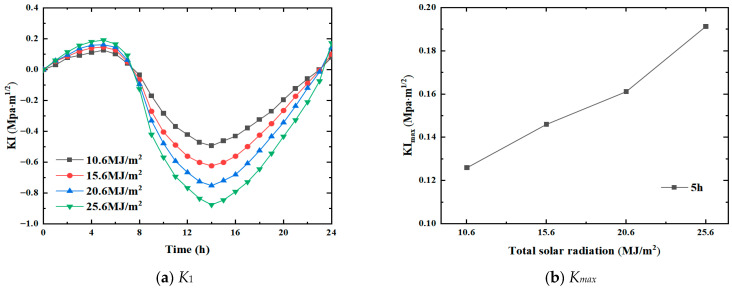
Variation trend under different solar radiations.

Since solar radiation is an essential part of heat conduction, the amount and intensity of solar radiation inevitably affect the propagation rate of transverse cracks in the pavement. In this study, the sunshine duration and wind speed were taken as c=8.4 h and v=3 m/s, respectively, while the total solar radiation was set to $Q=10.6\;MJ/m^{2}$, $15.6\;MJ/m^{2}$, $20.6\;MJ/m^{2}$, and $25.6\;MJ/m^{2}$. The crack propagation in the pavement under the defined solar radiation was explored. Figure 6 shows the variation trend of the mode I stress intensity factor under different solar radiations and corresponding positive peak values, respectively.

It is observed that the variation trend of the curves of the stress intensity factor is similar at all solar radiation intensities, i.e., it first increases, then decreases, and finally increases. However, the peak stress intensity factor at the tip of the transverse crack varies directly with the increase in the solar radiation intensity. In addition, the change in solar radiation does not significantly impact the stress state of the crack. The crack remains in the tensile opening from 0 h to 8 h and from 23 h to 24 h, and it tends to shrink under compression afterward.

As the total solar radiation changes, the stress intensity factor at the crack tip changes significantly. The positive peak at 5 h and the negative peak at 14 h change considerably. For the positive peaks, the corresponding stress intensity factor values are $0.126\;MPa\cdot m^{1/2}$ and $0.180\;MPa\cdot m^{1/2}$ for $Q=10.6\;MJ/m^{2}$ and $Q=25.6\;MJ/m^{2}$, respectively. For the negative peak, when $Q=10.6\;MJ/m^{2}$, the corresponding stress intensity factor K1 is $-0.494\;MPa\cdot m^{1/2}$; when $Q=25.6\;MJ/m^{2}$, the corresponding K1 is $-0.882\;MPa\cdot m^{1/2}$. The positive peak values of stress intensity factor Kmax under different solar radiations indicate that the growth of Kmax is nearly 50% as Q increases from 10.6 $MJ/m^{2}$ to 25.6 $MJ/m^{2}$. Therefore, for the same conditions, the larger the solar radiation, the greater the crack propagation.

### 4.2. Effect of Sunshine Duration on Transverse Crack Propagation in the Pavement

**Figure 7:**
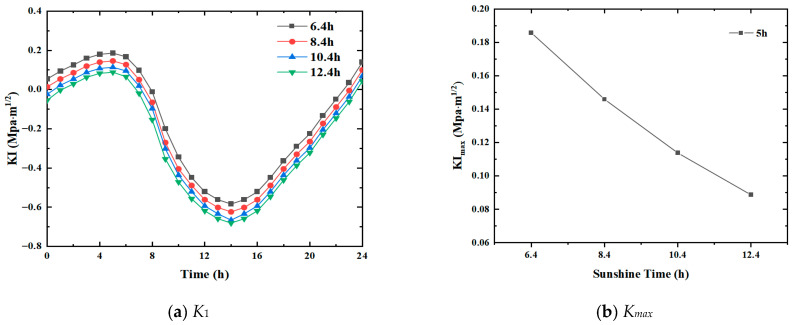
Variation trend under different solar exposure durations.

Since sunshine duration directly affects the solar radiation absorbed by the pavement, studying the effect of solar exposure duration on the transverse crack propagation in the pavement is imperative. In the subsequent analysis, the total solar radiation was fixed as $Q=15.6\;MJ/m^{2}$, while the wind speed was taken as v=3 m/s. Also, the sunshine duration was set to c = 6.4 h, 8.4 h, 10.4 h, and 12.4 h, respectively. Crack propagation in the pavement was then examined under these conditions. Figure 7 shows the variation in the mode I stress intensity factor and the maximum positive stress intensity factor under different solar exposure durations, respectively.

The variation in transverse cracks on the road surface is similar for all solar exposure durations considered, the only difference being the vertical shifts in curves. When the solar exposure duration is 6.4 h, the time for the crack to remain open under tensile stress exceeds 8 h. The time of crack opening gradually decreases with the increase in solar exposure duration. When the exposure duration is 12.4 h, the crack remains open under tension for less than 8 h, as shown in Figure 7b.

As the duration of solar exposure changes, the stress intensity factor at the crack tip drastically changes. The positive peak at 5 h and the negative peak at 14 h change significantly. For the positive peak, when c = 6.4 h, the corresponding stress intensity factor K1 is $0.186\;MPa\cdot m^{1/2}$, while when c = 12.4 h, K1 is only $0.092\;MPa\cdot m^{1/2}$. For the negative peak, the corresponding stress intensity factor K1 is $-0.585\;MPa\cdot m^{1/2}$ and $-0.678\;MPa\cdot m^{1/2}$ for when c = 6.4 h and c = 12.4 h, respectively. Kmax trend under various sunshine durations shows that as c increases from 6.4 h to 12.4 h, the positive amplitude is almost reduced by half. All other conditions being the same, the longer the duration of solar exposure, the smaller the crack propagation. This is because, as the duration of sunshine exposure increases, the pavement absorbs more heat, and the temperature change is reduced accordingly.

### 4.3. Effect of Wind Speed on Transverse Crack Propagation in the Pavement

**Figure 8:**
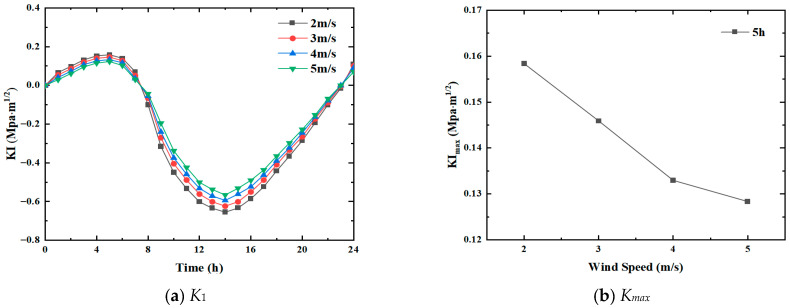
Variation trend under different wind speeds.

Heat exchange occurs between the road surface and the atmosphere, which is directly related to the heat transfer coefficient of the wearing surface. Since the heat transfer coefficient is linearly related to wind speed, the effect of wind speed on transverse crack propagation must be studied. In this analysis, the total solar radiation was fixed as $Q=15.6\;MJ/m^{2}$, while the solar exposure duration was taken as c = 8.4 h. As the wind speed was the parameter to be studied, it was set to v = 2 m/s, 3 m/s, 4 m/s, and 5 m/s, respectively. The corresponding crack propagation pattern of pavement under the changing wind speeds was obtained. Figure 8a shows the variation in the mode I stress intensity factor under varying wind speeds, while Figure 8b depicts the corresponding maximum positive stress intensity factor.

It is seen that with the increase in wind speed, the trend of the mode I stress intensity factor does not vary significantly. Before reaching the positive peaks, the K1 curves for different wind speeds are almost identical, and beyond the positive peak, the smaller the wind speed, the faster the decline in the curve. The time of crack opening does not change much with wind speed. The crack still tends to open from 0 h to 8 h and from 23 h to 24 h.

Although wind speed has a certain impact on stress intensity, it is not considerable compared to the corresponding values for solar radiation intensity and exposure duration. For the positive peak, when v = 2 m/s, the corresponding stress intensity factor K1 is $0.159\;MPa\cdot m^{1/2}$; while for v = 5 m/s, K1 is $0.128\;MPa\cdot m^{1/2}$. For the negative peak, the corresponding stress intensity factor K1 is $-0.655\;MPa\cdot m^{1/2}$ and $-0.565\;MPa\cdot m^{1/2}$, respectively, for when v = 2 m/s and when v = 5 m/s. Kmax values indicate a decreasing trend with increasing wind speeds. As v increases from 2 m/s to 5 m/s, the decrease is about 20%. Hence, all other conditions being equal, the greater the wind speed, the smaller the crack propagation.

## 5. Crack Propagation Under Cyclic Temperature Load in Winter

In addition to top-down surface cracks, bottom-up reflective cracks often appear in the base layer of asphalt pavements in winter. After reflective crack formation in the pavement base layer, stress concentration occurs under cyclic temperature loads. However, the cracks do not propagate immediately since the material has a certain strength. As the number of cycles continues to increase, a certain amount of fatigue damage occurs inside the material, and according to the Boltzmann superposition principle, the damage continues to accumulate with the increase in the number of cycles. With the continuous aging of the material and its degradation due to external factors, such as rain and snow, the tensile strength of the material continues to decrease, eventually leading to crack propagation and failure.

### 5.1. Developing the Two-Dimensional XFEM Model

**Figure 9:**
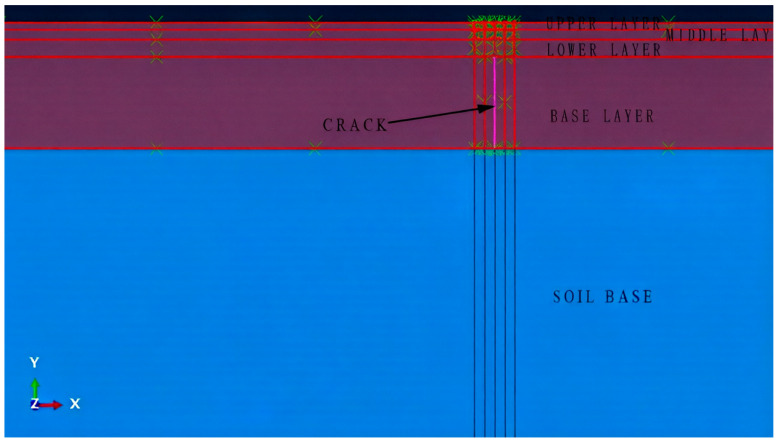
Two-dimensional finite element model and crack location.

In analyzing the mode I stress intensity factor presented earlier, three-dimensional finite element modeling is employed to restore the actual situation as much as possible. However, introducing the relevant parameters of the Paris equation and adopting the direct cyclic approach is critical while studying crack propagation under cyclic temperature load in winter. The Paris equation uses the Fourier method, and many conventional modeling settings are not consistent with this approach. If the model is too complex on the macroscopic level, it is also less effective for fatigue analysis [31,32]. Therefore, the two-dimensional FE method was adopted to analyze the fatigue crack propagation of semi-rigid base asphalt pavement under cyclic temperature loading. The aforementioned three-dimensional model was simplified into a two-dimensional one, and a crack running through the base layer was set up. Maxps damage was selected as the damage criterion. The crack initiation and propagation were determined according to the maximum principal stress and fracture energy. The specific model is presented in Figure 9.

### 5.2. Establishment of the Cyclic Temperature Field

**Figure 10:**
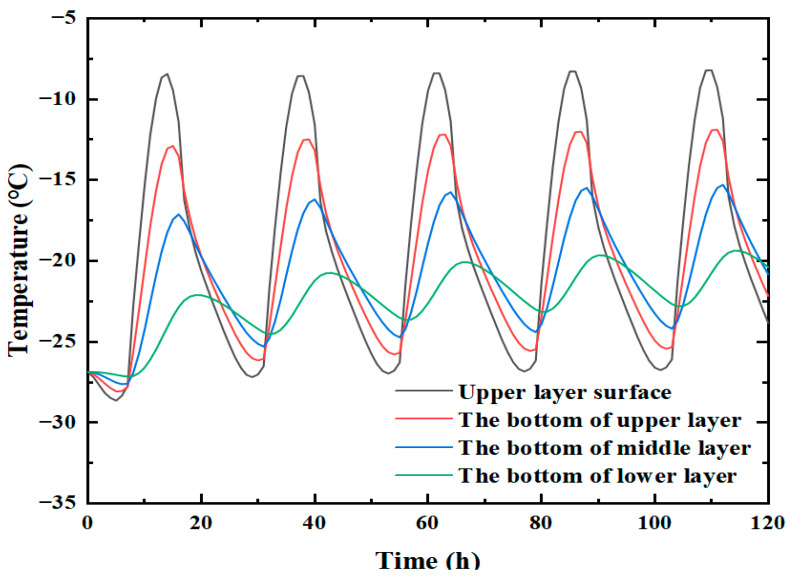
Temperature curves of each layer at 120 h in the circulating temperature field.

The temperature cycle was modified from the original setting of a fixed 24 h to a continuous 24 h cycle. From the results discussed earlier, it can be seen that for the temperature parameters, the crack is in compression shrinkage for most of the day, and it is less likely for the crack to propagate. In order to better observe the crack propagation pattern under cyclic temperature load, a new cyclic temperature field featuring a larger temperature difference and a temperature below 0 °C throughout the day was introduced herein. This temperature field is more conducive to crack propagation. In addition, all the other parameters are the same as those mentioned above, as shown in Figure 10.

### 5.3. Fatigue Propagation of Reflective Cracks

**Figure 11:**
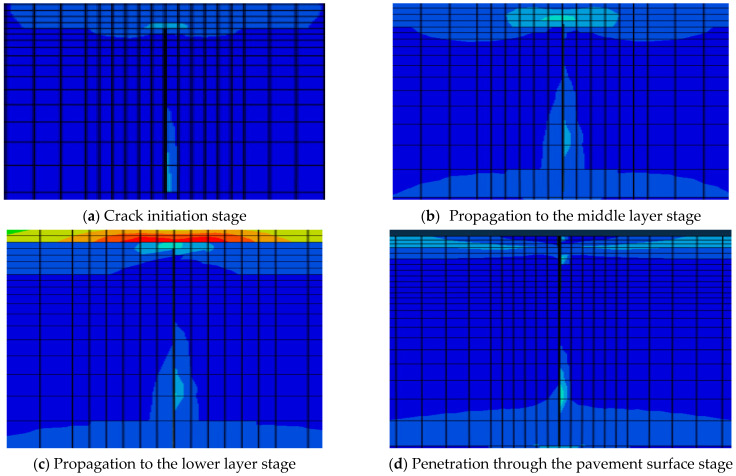
Stress distribution during the propagation of the reflective crack.

**Figure 12:**
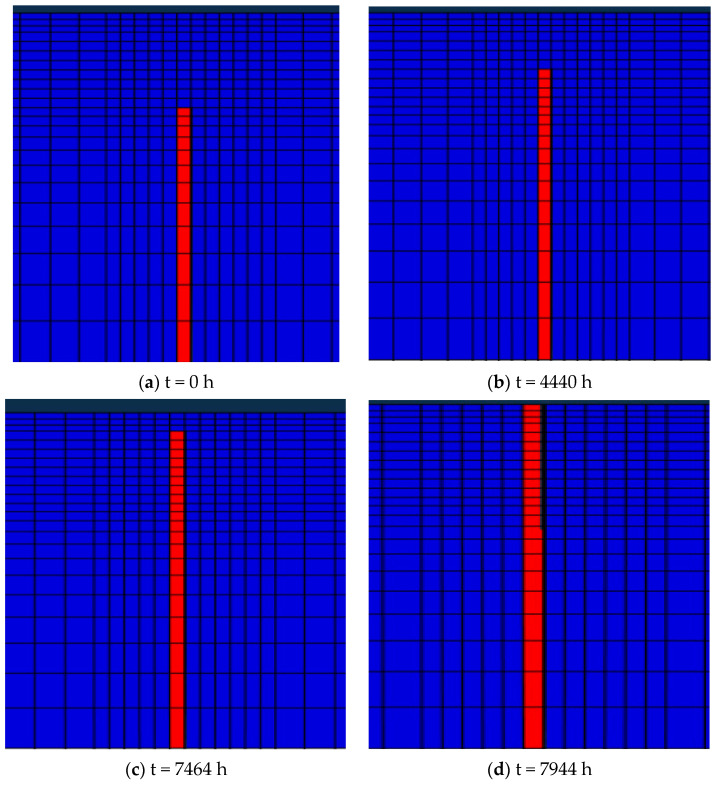
Expansion path of the reflective crack.

Figure 11 shows the stress distribution when the reflective crack in the base layer propagates under the cyclic temperature field depicted in Figure 11. As the crack is about to propagate at the initial stage, the stress concentration occurs at the crack tip, as shown in Figure 12a. Figure 12b shows the crack initiation process when the crack meets the propagation condition, while the continuous upward propagation and extension of the crack are shown in Figure 12c. Figure 12d shows the process of the crack finally penetrating the surface layer and disrupting the road surface.

In ABAQUS, the crack propagation path can be viewed through damage. Figure 12 shows the crack damage propagation over time.

**Figure 13:**
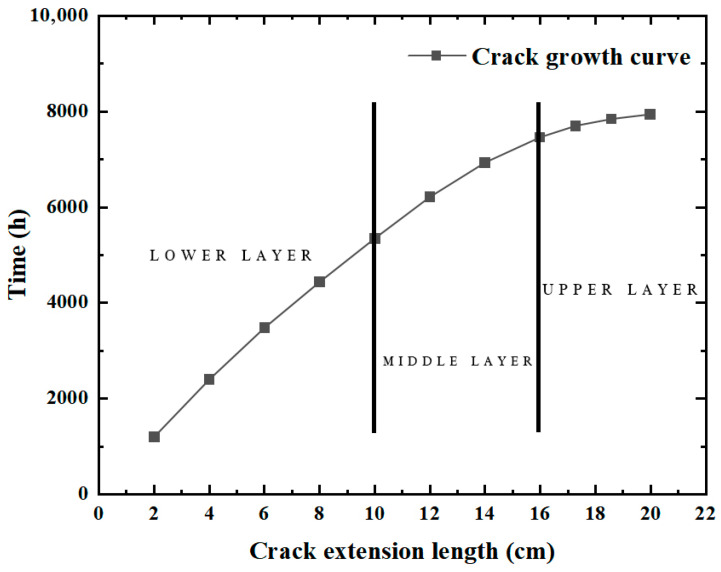
Relationship between crack length and time.

The crack expansion process is shown in Figure 13. The crack length increases with the loading time. In the early stage of crack propagation, the crack propagation is slow because the temperature change at the bottom of the surface layer is much less than that of the surface layer. The crack begins to propagate only when t = 1200 h. However, as the crack continues to propagate upward, the temperature stress rises, and the crack propagation rate increases rapidly. It can be seen that it takes 5352 h for the crack to penetrate the lower layer, i.e., when the crack length increases to 10 cm. Likewise, it takes 2112 h for the crack to penetrate the middle layer, with the crack growing from 10 cm to 16 cm, whereas it merely takes 480 h for the crack to penetrate the upper layer, as the crack length increases from 16 cm to 20 cm.

### 5.4. Effect of Initial Crack Length

**Figure 14:**
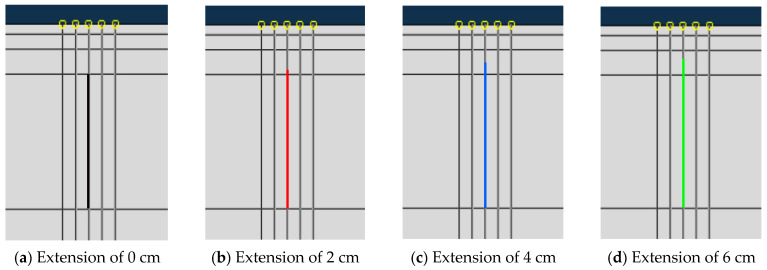
Initial crack length.

In the analysis, the crack is considered only in the base layer. However, in actual scenarios, the initial cracks often exist not only in the base layer but sometimes extend to the part of the surface layer. In this section, while keeping other conditions unchanged, the initial crack was extended by 0 cm, 2 cm, 4 cm, and 6 cm, respectively, and the corresponding effect on crack propagation was evaluated. The initial crack lengths are shown in Figure 14.

**Figure 15:**
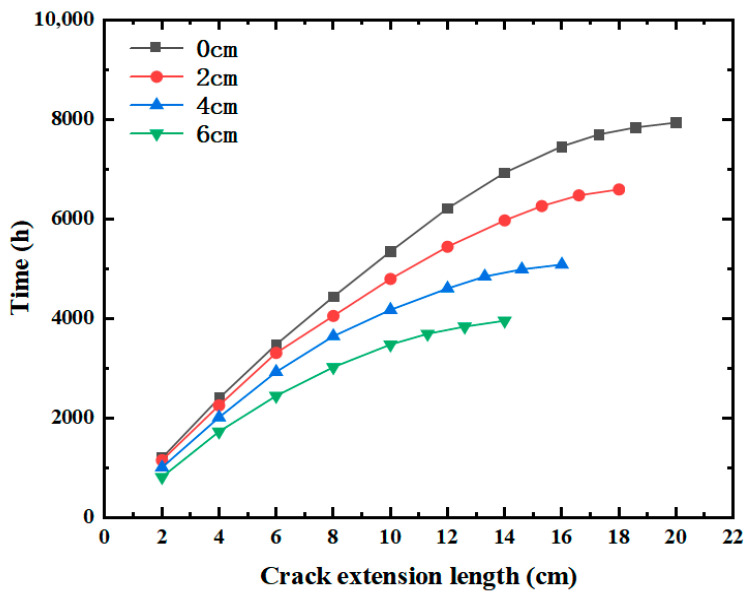
Effect of initial crack length.

Figure 15 shows the propagation time of cracks with different initial lengths through the surface layer. It is observed that the initial crack length does not affect the overall trend of the crack propagation. Irrespective of the initial crack length, the crack length grows slowly and then rapidly over time. However, the initial crack length can significantly affect the pavement service life. As the initial crack length increases, the pavement life decreases substantially. When the initial crack length is extended by 0 cm, the crack propagation time to the surface layer is t = 7944 h. When the initial crack length is extended by 6 cm, the crack propagation time to the surface layer is t = 3960 h, i.e., a decline of nearly 50%. It is also seen that the longer the initial crack length, the faster the crack propagation, primarily because the tip of the longer crack is closer to the road surface and the temperature difference is larger, so the resulting temperature stress will also be greater, facilitating crack propagation.

### 5.5. Effect of Initial Crack Angle

**Figure 16:**
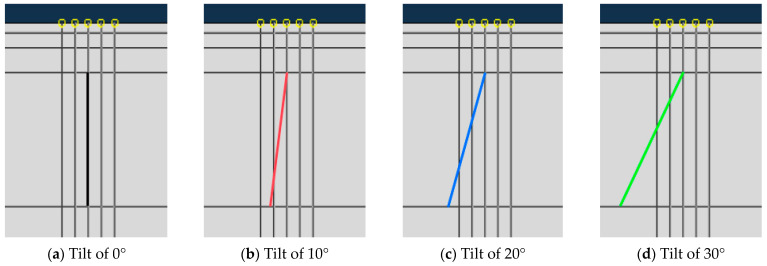
Initial crack angle.

In actual situations, cracks are almost never completely perpendicular to the road surface, and all of them present at a certain inclination angle with the road surface. Therefore, in this section, the influence on crack propagation under the condition that the inclination angle is 0°,10°, 20°, and 30° is explored, respectively. The specific schematic diagram of the crack inclination angle is shown in Figure 16.

**Figure 17:**
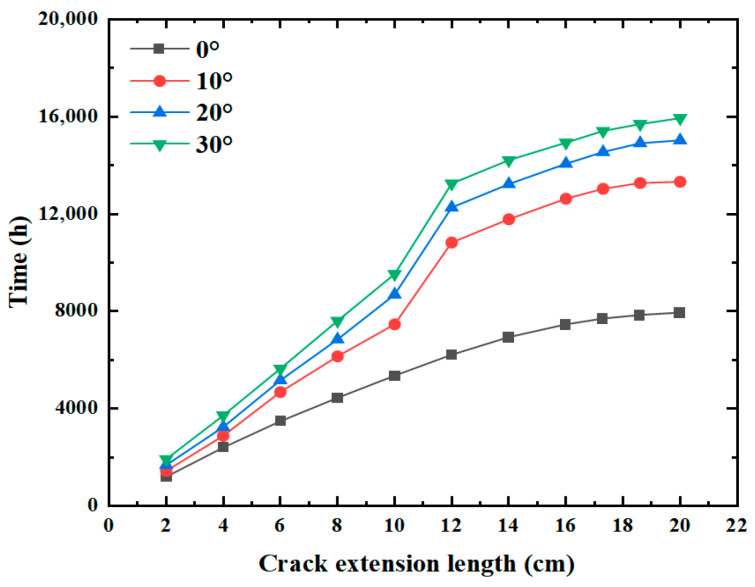
Effect of initial crack angle.

According to the analysis of the time taken by the crack to expand to the surface layer under different initial inclination angles in Figure 17, it can be found that the change in the initial fracture inclination angle will have a certain influence on the overall change trend of the crack. When the inclination angle is 0°, the fracture propagation length increases slowly first and then rapidly with the passage of time. When the crack has a certain inclination angle, it will grow very slowly to a certain length. For example, when the expansion length is between 10 and 12 cm in the figure, it can be obviously observed that the crack grows very slowly, mainly because the strength of the middle surface layer is high, and the crack needs to provide more force to fully penetrate the crack. But after that time, the cracks began to grow rapidly again. In addition, it can be seen from the images that the inclination angle of the initial crack will significantly affect the life of the pavement. With the increase in the initial inclination angle of the crack, the life of the pavement will increase significantly. When the initial inclination angle of the crack is 0°, the time taken by the crack to extend to the surface layer is t = 7944 h; when the initial inclination angle of the crack is 30°, the time taken by the crack to extend to the surface layer is t = 15,936 h. It can be seen that the larger the initial crack inclination angle, the slower the crack expansion, mainly because the crack tip expansion path with the initial inclination angle is not vertical upward, but has a certain angle with the road surface, which leads to the actual distance of the crack expansion to the road surface is oblique, the distance is longer, so the crack is more difficult to expand.

## 6. Conclusions

In this paper, the influence of the temperature field on crack propagation is studied by FE analysis. The following conclusions are drawn from the obtained results.

(1) During a day, the temperature variation field inside the pavement has a sinusoidal trend. The closer to the road surface, the more drastic the temperature change. The temperature gradient, combined with the differences in the coefficients of thermal expansion among structural layers, jointly induces crack opening.
(2) Changes in the temperature field mainly cause cracking in opening mode (mode I). The variation in the mode I stress intensity factor in winter is almost twice that in summer, so the opening mode in winter conditions is the most unfavorable cracking scenario.
(3) During the daily temperature variation, the total solar radiation intensity, duration of solar exposure, and wind speed all influence crack propagation. Specifically, greater total solar radiation leads to increased crack propagation, whereas higher wind speed results in reduced crack propagation. Similarly, longer exposure duration corresponds to a smaller extent of crack propagation.
(4) Under the cyclic temperature field, the length of the reflective crack is directly proportional to time. An increase in the initial crack length significantly reduces the service life of pavement, while an increase in the initial crack inclination angle can markedly extend pavement’s service life.

In this study, all models were configured with only a single crack. However, in real pavement, multiple cracks inevitably exist due to various influencing factors. According to relevant literature, interactions occur among multiple cracks. Therefore, future work should investigate the propagation behavior of multiple cracks under combined thermal and vehicle loads.

## Figures and Tables

**Table 1:** Thermodynamic parameters of materials for each structural layer of the pavement.

Description	AC-13	AC-20	ATB-25	Cement-Stabilized Macadam Base Layer	Soil Base
Thermal conductivity $k/\left(J/m\;h\right)$	4680	5040	4320	5616	5616
Thickness/cm	4	6	10	54	--
Density $\rho /\left(kg/m^{3}\right)$	2300	2400	2400	2200	1800
Specific heat C/(J/kg·K)	924.9	900	815	911.7	1040
Solar radiation absorptivity $a_{s}$	0.9				
Pavement emissivity ε	0.81				
Absolute zero $T_{z}/{}^{\circ}C$	−273				
Stefan-Boltzmann constant $\sigma /\left(J/h\cdot m^{2}\cdot K^{4}\right)$	0.0002041092				

Note: AC-13 refers to asphalt concrete with a maximum nominal aggregate size of 13 mm. AC-20 refers to asphalt concrete with a maximum nominal aggregate size of 20 mm. ATB-25 refers to an asphalt-treated base layer with a maximum nominal aggregate size of 25 mm.

**Table 2:** Parameters of summer temperature variation (July).

$Q=26.3\;MJ/m^{2}$		c=10.7 h		v=2.6 m/s	
Duration (h)	Temperature (°C)	Duration (h)	Temperature (°C)	Duration (h)	Temperature (°C)
1	24.9	9	29.3	17	33.7
2	23.8	10	31.3	18	32.6
3	23.3	11	33.1	19	31.6
4	23.1	12	34.5	20	30.4
5	22.9	13	35.3	21	29.5
6	24.2	14	35.7	22	28.1
7	25.4	15	35.4	23	26.9
8	27.3	16	34.6	24	25.6

**Table 3:** Parameters of winter temperature variation (January).

$Q=15.6\;MJ/m^{2}$		c=8.4 h		v=3 m/s	
Duration (h)	Temperature (°C)	Duration (h)	Temperature (°C)	Duration (h)	Temperature (°C)
1	−1.4	9	3.3	17	7.8
2	−2.4	10	5.3	18	6.7
3	−3.0	11	7.1	19	5.6
4	−3.1	12	8.5	20	4.4
5	−3.3	13	9.4	21	3.4
6	−2.1	14	9.7	22	2.2
7	−0.7	15	9.4	23	0.8
8	1.3	16	8.8	24	−0.4

**Table 4:** Thermal expansion coefficients of each structural layer of the pavement at different temperatures.

Structural Layer	Temperature (°C)								
	−15	−10	0	10	15	20	25	30	40
Upper layer	3.2	3.8	4.3	3.5	3.2	2.9	2.6	2.3	1.97
Middle layer	1.8	2.1	2.6	2.4	2.1	1.8	1.5	1.2	1.0
Lower layer	1.7	1.8	2.2	2.0	1.8	1.6	1.4	1.2	1.0
Base layer	0.98								
Soil base	0.45								

**Table 5:** Prony series of the surface layer of the asphalt pavement.

$\tau_{i}(s)$	0.00001	0.0001	0.001	0.01	0.1	1	10	100	1000
Upper layer	0.1007	0.1039	0.1773	0.2356	0.2003	0.1036	0.0401	0.0142	0.0074
Middle layer	0.1679	0.1793	0.2555	0.2194	0.1049	0.0368	0.0129	0.0048	0.0022

Note: The Prony series is a set of parameters used to describe the viscoelastic behavior of materials, and it is not a physical quantity with a single unit of measurement.

**Table 6:** Relevant parameters of the asphalt pavement.

Structural Layer	Thickness/m	Modulus/MPa	Poisson’s Ratio	Density/(kg/m^3^)
Upper layer	0.04	7500	0.25	2400
Middle layer	0.06	9000	0.25	2400
Lower layer	0.1	7000	0.25	2400
Base layer	0.54	9000	0.25	2300
Soil base	2.26	400	0.4	1800

**Table 7:** Relevant parameters of the WLF equation of the surface layer of the asphalt pavement.

Material	$T_{0}/{}^{\circ}C$	$C_{1}$	$C_{2}$
Upper layer	20	33.5	284.9
Middle layer	20	30.9	272.4

## Data Availability

The original contributions presented in this study are included in the article. Further inquiries can be directed to the corresponding author.
